# Role of antibiotic envelopes in preventing cardiac implantable electronic device infection: A meta‐analysis of 14 859 procedures

**DOI:** 10.1002/joa3.12302

**Published:** 2020-01-16

**Authors:** Abdul Aziz A. Asbeutah

**Affiliations:** ^1^ Division of Cardiology Beth Israel Deaconess Medical Center Harvard Medical School Boston MA USA

To the Editor,

I read with great interest the meta‐analysis by Kumar et al^1^ entitled “Role of antibiotic envelopes in preventing cardiac implantable electronic device infection: A meta‐analysis of 14 859 procedures.” Their primary analysis demonstrates a 59% reduction in major/minor risk of infection in favor of the antibiotic envelope (Risk Ratio, 0.41; 95% Confidence Interval, 0.31‐0.54). They also performed a subgroup analysis of two studies, according to the authors, that used an absorbable envelope compared to no envelope in the prevention of device‐related infections and showed a significant 52% reduction in risk of major/minor device‐related infections (Risk Ratio, 0.48; 95% Confidence Interval, 0.35‐0.65). Therefore, I believe that this study should not be included in this meta‐analysis as it did not include any true device‐related infections as it was based on a predictive model by gathering data from several other studies (Risk ratio, 0.16; 95% confidence interval, 0.08‐0.35). The study by Kay et al^2^ did not report any true device‐related infections in their patients as it was a cost‐effectiveness analysis using infection rate data from previously published studies^3^, ^4^, ^5^, included in this meta‐analysis, and extrapolated it to their patient population in the United Kingdom National Health Services based on a predictive model. Therefore, I believe that this study should not be included in this meta‐analysis as it did not include any true device‐related infections and it was based on a predictive model by gathering data from several other studies.

Additionally, the authors included a retrospective study by Kolek et al^5^ that aimed to study the efficacy of an antibiotic envelope in the prevention of cardiac implantable electronic device (CIED) infections in high‐risk patients. This study by Kolek et al^5^ included 353 patients who received nonabsorbable antibiotic envelopes, 135 patients who received absorbable envelopes, and 636 patients that did not receive an envelope. However, authors of this meta‐analysis only included subjects who received a nonabsorbable envelope in their analysis. Albeit technically difficult to include the absorbable envelope group from Kolek et al^5^ in the analysis due to overlap of subjects in the control group, it is worth mentioning this in the discussion or limitations of the manuscript. In summary, the results of this meta‐analysis support the benefit of an antibiotic envelope in the reduction of device‐related infections in patients undergoing CIED implantation or upgrade; however, an accurate summary of the effect depends on the correct selection of studies to include for analysis.

## CONFLICT OF INTEREST

Authors declare no conflict of interests for this article.
